# Effect of Endophyte Infection and Clipping Treatment on Resistance and Tolerance of *Achnatherum sibiricum*

**DOI:** 10.3389/fmicb.2016.01988

**Published:** 2016-12-15

**Authors:** Junhua Qin, Yuan Gao, Hui Liu, Yong Zhou, Anzhi Ren, Yubao Gao

**Affiliations:** College of Life SciencesNankai University, Tianjin, China

**Keywords:** *Achnatherum sibiricum*, clipping, endophyte, locust, resistance, tolerance

## Abstract

It is well-documented that endophytes can enhance the resistance of agronomical grasses, such as tall fescue and perennial ryegrass to herbivory. For native grasses, however, the related reports are limited, and the conclusions are variable. *Achnatherum sibiricum* is a grass native to the Inner Mongolian steppe. This grass is highly infected by endophytes but does not produce detectable endophyte-related alkaloids known under normal conditions. In this study, the contributions of endophytes to the resistance of *A. sibiricum* to *Locusta migratoria* were studied. We found that locusts preferred EF (endophyte-free) plants to EI (endophyte-infected) plants, and the weight of locusts fed on EI plants was significantly lower than those fed on EF plants. Hence, endophyte infection significantly enhanced the resistance of the host to *L. migratoria*. Endophyte infection significantly decreased the concentration of soluble sugar and amino acids while significantly increased the concentration of total phenolic content, and these metabolites may contribute to herbivore resistance of the host. The clipping treatment further strengthened the locust resistance advantage of EI over EF plants. After clipping, the weight of the locusts fed on EI plants significantly decreased compared with those fed on unclipped plants, whereas the weight of the locusts fed on EF plants increased significantly. The results suggested that endophyte infection could increase herbivore resistance while decreasing the tolerance of the host grass by mechanisms apart from endophyte-conferred alkaloid defense.

## Introduction

Plants can coexist with many types of microbial symbiosis, such as rhizosphere bacteria, mycorrhizal fungi and endophytes. Endophytes live asymptomatically within many cool season grasses for at least a portion of their life cycle (Carroll, 1988). Thus far, the best-studied endophytes are *Neotyphodium lolii* and *Neotyphodium coenophialum*, which colonize perennial ryegrass (*Lolium perenne* L.) and tall fescue (*Lolium arundinaceum* Darbyshire ex. Schreb.), respectively. Pioneering studies by Prestidge et al. (1982) reported that endophyte infection could increase the resistance of tall fescue to *Listronotus bonariensis* (Argentine stem weevil); since then, more studies on the increased herbivore resistance to endophyte infection have been reported in cultivated grasses, including species of *Festuca*, *Lolium*, and *Poa* (Siegel et al., 1990; Bultman and Conard, 1998; Kunkel et al., 2004). The anti-herbivore properties of endophyte infection are largely attributable to the production of a variety of alkaloids (Schardl et al., 2004; Bush and Fannin, 2009). Thus far, four classes of alkaloids associated with infected host grasses have been detected, including saturated aminopyrrolizidines (lolines), pyrrolopyrazines (peramines), ergot alkaloids and indolditerpenes (lolitrems) (Bush et al., 1997; Schardl et al., 2012). Lolines have a broad spectrum of activity against insects (Bultman et al., 1997). Peramine is known to act as a feeding deterrent (Prestidge and Gallagher, 1988). Ergot alkaloids are primarily active against vertebrates, whereas lolitrems are responsible for neurotoxic disorders of mammals (Prestidge, 1993).

Endophytes not only exist in cultivated grasses such as tall fescue and perennial ryegrass but are also widely distributed in native grasses (Leuchtmann, 1993). There are a variety of native grass hosts, and the circumstances of endophyte infection are more complicated in native grasses (Afkhami and Rudgers, 2009; Faeth and Saari, 2012). In contrast with cultivated grasses, endophytes in native grasses usually produce fewer classes and a lower concentration of alkaloids (Faeth et al., 2002). Some endophytes only produce peramine, and some do not even produce alkaloids (Leuchtmann et al., 2000). Can endophyte infection in native grasses, which do not produce alkaloids or produce a low concentration of alkaloids, enhance the herbivore resistance of host plants? The limited interactions reported are highly variable. In a feeding assay with the leaf material of *Brachypodium sylvaticum*, Brem and Leuchtmann (2001) found that insect larvae performed significantly better on a diet of uninfected leaves. Lopez et al. (1995) found that *Melanoplus femurrubrum* consumed similar amounts of Arizona fescue (*Festuca arizonica*) regardless of endophyte status. Faeth and Shochat (2010) found that endophyte-infected (EI) Arizona fescue harbored more herbivorous insects than uninfected plants, suggesting that endophyte infection decreases rather than increases resistance to herbivores.

*Achnatherum sibiricum* (L.) Keng is a caespitose perennial grass that is widely distributed in northern China and commonly infected by *Epichloë* endophyte (Wei et al., 2006). Within the genus *Achnatherum* there are five sections, and *A. sibiricum* belongs to section Achnatheropsis (Tzvel.) N. S. Probatova. In this section there are nine species, including seven Asian and two American species (Wu and Lu, 1995). Before *A. sibiricum*, two other species, *Achnatherum inebrians* and *Achnatherum robustum*, have been reported for their narcotic effects on livestock resulting from endophyte infection, and hence are known as ‘drunken horse grass’ and ‘sleepy grass,’ respectively (Petroski et al., 1992; Bruehl et al., 1994). In contrast, *A. sibiricum* exhibits no obvious herbivore deterrence ability according to local records and our own observations. *A. sibiricum* is infected by two species of endophytes, *Epichloë gansuensis* and *Epichloë sibirica*, in its native populations (Zhang et al., 2009; Li et al., 2015). In the Inner Mongolian steppe, locusts are the primary consumer, and they affect grassland productivity and compete with domestic animals for food resources (Kang et al., 2007). The biogeography of 150 species of locust fauna on the Inner Mongolian Plateau has been studied, of which 10–15 species are considered as grassland pests (Kang et al., 2007). *Locusta migratoria* (Orthoptera: Acrididae) is oligophagous, feeding mainly on grasses of Gramineae and Cyperaceae (Bernays et al., 1976). It is famous for its wide breeding range, strong stress resistance and fecundity, and it can also have a long-distance migration.

In our previous investigation, we found that alkaloids associated with endophyte infection were detected in neither infected nor uninfected plants when grown under normal conditions. After clipping, only peramine was detected, but its concentration in the sheath of infected plants ranged from 0 to 0.6 ppm. In this study, EI *A. sibiricum* was adopted as plant material. Here, endophyte infection instead of endophyte species was considered. *L. migratoria*, a common herbivore of grasses (Yue et al., 2009), was adopted as the feeding herbivore. Clipping is a common practice in our sampling grassland; thus, we use clipping as the interference. We wondered whether endophyte infection has a positive effect on the insect resistance of *A. sibiricum* and whether clipping can influence insect resistance of infected *A. sibiricum*. Furthermore, how do the strategies of infected and uninfected *A. sibiricum* respond to clipping?

## Materials and Methods

### Seed Source

Seeds of *A. sibiricum* were collected from the National Hulunber Grassland Ecosystem Observation and Research Station (49.06° N, 119.40° E) in 2012. After detection, we found that the endophyte infection rate was 100%. To obtain endophyte-free (EF) seeds, EI seeds were placed in a 60°C oven for 30 days. The previous study in our lab showed that a high temperature treatment for 30 days could completely destroy endophytes in the seeds and that it had no significant influence on seed germination rate, germination potential, and germination index (Li et al., 2010); a similar method to obtain EF seeds was also reported by Kannadan and Rudgers (2008) in grove bluegrass.

### Locusta migratoria

*Locusta migratoria* is not a dominant species in the Inner Mongolia steppe. It was chosen as an herbivore because it is known to cause significant damage to grasses and because it is readily available and easily cultured. Eggs were purchased from a local pet shop. After hatching unearth eggs in an oven containing moist vermiculite for approximately 2 weeks in the dark at 25°C, the nymphs were placed in a special device, which was conducted in a constant temperature room at 25°C with a 12 h light /12 h dark photoregime, and fed with wheat seedlings. After growing into the requisite instars (second instar or fourth instar), the nymphs were used in the experiments.

### Plant Treatment

The plants used in this experiment were obtained either from EI seeds or EF seeds. The seeds were planted in white plastic pots (20 cm in diameter and 20 cm in depth) filled with vermiculite. After 10 days’ growth, 20 plants of approximately equal size were maintained in each pot. After 5 weeks of cultivation, the plants in half pots were clipped with scissors 5 cm above the soil surface, and the other half was retained as a control (CK, unclipping). All seedlings were separated into four groups, i.e., EI-CK, EI-Clipping, EF-CK, and EF-Clipping. The plants were cultivated for a further 3 weeks before the locust feeding experiment was performed. Each group comprised 10 replicates, with five replicates for the locust feeding experiment and the other five replicates for the sampling and measurement of physiological characteristics. The experimental plants were watered and fertilized with Hoagland nutrient solution as needed. The experiment was conducted in the greenhouse at Nankai University, Tianjin, China.

### Choice Feeding Experiment

After 24 h’ starvation, fourth instar nymphs of the locusts were transferred to transparent plastic containers of a 19 cm height and a 8 cm diameter. As food, 0.5 g leaf blades per pot per treatment were cut and offered in small glass dishes, separately. 0.5 g plant material was sufficient for the locusts feeding in 1 h to avoid being depleted completely during the trial. There were 10 plastic containers in total. Eight locusts were added to each of five containers. Another five containers were used as a control to calculate the reduced leaf quality due to evaporation. After 1 h of exposure to herbivory, all plants from all containers were harvested, and the fresh biomass was recorded.

### Nymphs’ Growth Experiment

We equipped every pot with a steel frame of 45 cm height and 20 cm diameter which was coated with a nylon stocking. To launch the experiment, three weighed second instar nymphs of the locust hatchlings from one population were introduced into each of these resulting cages. There were 20 pots in total, including EI-CK, EI-Clipping, EF-CK and EF-Clipping, and each treatment was performed for five replicates. The experiment lasted for 5 days, and the plant material was adequate for the second instar locusts. In the end, the biomass of the nymphs was weighed and recorded.

### Quantification of Amino Acid Concentrations

The amino acids in the shoot were analyzed by reverse-phase high-performance liquid chromatography (HPLC) with pre-column derivatization using 2, 4-dinitro-fluorobenzene (DNFB) according to Li and Sun (2004). The standard solutions were AA-S-18 (Sigma-Aldrich) and stored at 2–8°C. The analysis was performed using a Wasters HPLC system (Waters 1500-series). A reverse-phase C18 column (5 μm, 150 mm × 4.6 mm) and fluorescence detector were used for the chromatographic separation. The column was maintained at 25°C with a gradient (1 ml min^-1^ flow) programmed as follows: 84/16 (6 min),76/24 (6 min), 70/30 (6 min), 60/40 (12 min), 50/50 (7 min), 2/98 (5 min), 20/80 (8 min), 84/16 (5 min), and 84/16 (5 min holding) of eluent A/eluent B. Eluent A comprised 50% acetonitrile and 50% water. Eluent B comprised 37.5 mmol L^-1^ NaAc, 10% acetonitrile, and 1% *N,N*-Dimethyl-formamide (with a pH of 6.4 adjusted with glacial acetic acid).

### Other Variables Measured

The soluble sugar content was analyzed according to Buysse and Merckx (1993) and Yu et al. (2011). The total phenolic concentration was determined according to Malinowski et al. (1998).

### Root Morphology and Plant Regrowth Rate

Fresh roots were washed and then scanned with an EPSON 1680 scanner (Epson, Long Beach, CA, USA) in 400 dpi resolution. After scanning, the root image was analyzed with WinRHIZO 2012 software to obtain parameters such as root length, root surface area and root average diameter. To assess the effect of endophyte infection on the regrowth ability of *A. sibiricum* after clipping, we grew additional EI (*n* = 5) and EF (*n* = 5) plants in white plastic pots, treated as before, and the plants were clipped 5 cm above the soil surface after 5 weeks of growth. The removed shoot tissue was oven dried and weighed. The plants were allowed to regrow for 3 weeks, at which time they were clipped at the soil surface. The shoot material was oven dried and weighed. The rate of regrowth of the plants was calculated as (ln[final mass]-ln[initial mass])/21 days (Bultman et al., 2004).

### Statistical Analysis

For the amino acids, we performed a principal components analysis (PCA) on the correlations among the 17 response variables and then performed factor rotation using the varimax method (Rasmussen et al., 2008; Guo et al., 2014). A varimax rotation is a change of coordinates that maximizes the sum of the variances of the squared loadings. This method increases the distinction between the large and small loading variables and so makes the biological interpretation of the axes simpler (Rasmussen et al., 2008). After varimax rotation, we retained four rotated factors (RFs). The RF variables were subjected to a two-way ANOVA with endophyte and clipping treatment as the factors. Other indexes were analyzed using multivariate and univariate analyses of variance. All analyses were performed using SPSS 21.0 software. The effects were considered significant if *P* < 0.05.

## Results

### Bioassay

**FIGURE 1:**
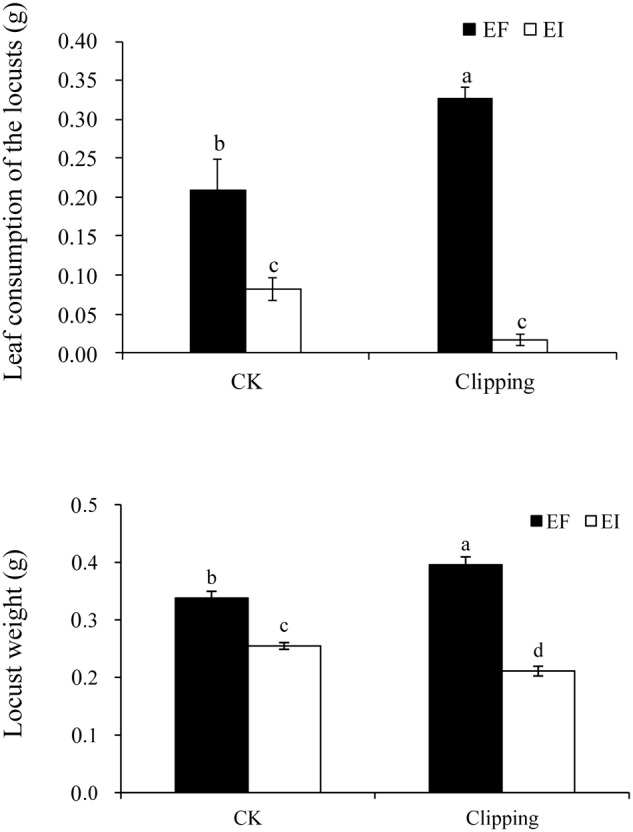
**Comparison of leaf consumption of endophyte-infected (EI) or endophyte-free (EF) *Achnatherum sibiricum* by the locusts and the weight of the second instars locusts fed on EI or EF plants under unclipping (CK) and clipping treatments.** Different lower-case letters denote means that are significantly different (*P* < 0.05).

In the choice experiment, the performance of *L*. *migratoria* was significantly influenced by endophyte infection and the interaction of endophyte infection and clipping (**Table 1**). Locusts preferred EF plants to EI plants. After clipping, EF plants were fed on more by locusts than the unclipped control plants. For EI plants, however, the biomass fed by locusts tended to be less with clipping treatment, but the difference was not significant. Thus, the difference of the biomass fed on by locusts between EI and EF plants were more obvious after clipping (**Figure 1**).

**Table 1:** Two-way ANOVA for leaf consumption and physiological indexes of endophyte-infected (EI) and endophyte-free (EF) *Achnatherum sibiricum* under the clipping treatment.

Treatment	Leaf consumption		Weight of the locusts		Soluble sugar in the shoot		Total phenolic content in the shoot		Total phenolic content in the root		Total root length		Total root surface area		Average root diameter	
	*F*	*P*	*F*	*P*	*F*	*P*	*F*	*P*	*F*	*P*	*F*	*P*	*F*	*P*	*F*	*P*
Endophyte (E)	89.26	**0.000**	165.7	**0.000**	67.07	**0.000**	21.21	**0.000**	51.60	**0.000**	1.370	0.259	0.091	0.767	2.935	0.106
Clipping (C)	1.306	0.270	0.473	0.501	0.001	0.979	52.40	**0.000**	50.10	**0.000**	245.2	**0.000**	17.75	**0.001**	155.5	**0.000**
E **×** C	15.67	**0.001**	23.87	**0.000**	11.57	**0.004**	6.235	**0.024**	4.725	**0.045**	11.31	**0.004**	0.950	0.344	5.636	**0.030**

*Significant P-values (P < 0.05) are in bold.*

In the growth experiment on the nymphs, the weight of the second instar locusts was significantly influenced by both endophyte infection and the interaction of endophyte infection and clipping (**Table 1**). Endophyte infection negatively affected the weight of *L*. *migratoria* nymphs. In the control group, the weight of the locusts fed by EI plants was significantly less than those fed by EF plants. After clipping, the weight of the locusts fed on EI plants decreased more than those fed on the unclipped control while the weight of the locusts fed on EF plants increased. Thus, the difference between the weights of the nymphs fed on the EI and EF plants was more obvious after clipping (**Figure 1**).

### Amino Acid Concentration in Plants

**FIGURE 2:**
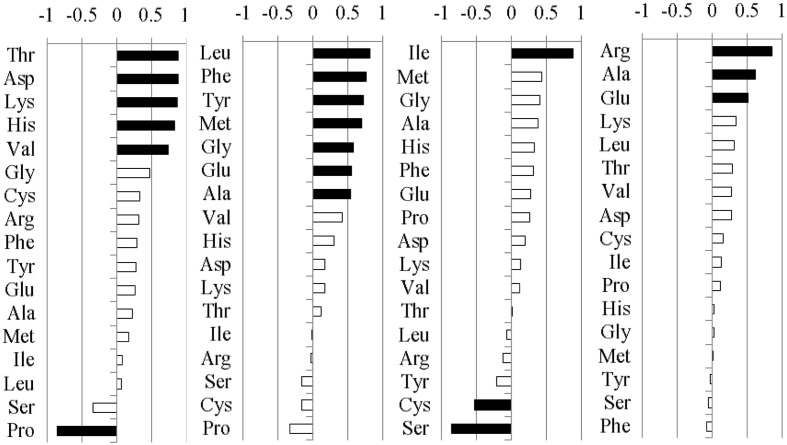
**Loadings for each individual amino acid of EI or EF *A. sibiricum* onto the first four rotated factors (RFs).** The individual amino acids loading heavily either positively (loading ≥ +0.5) or negatively (loading ≤ -0.5) are highlighted in black.

**FIGURE 3:**
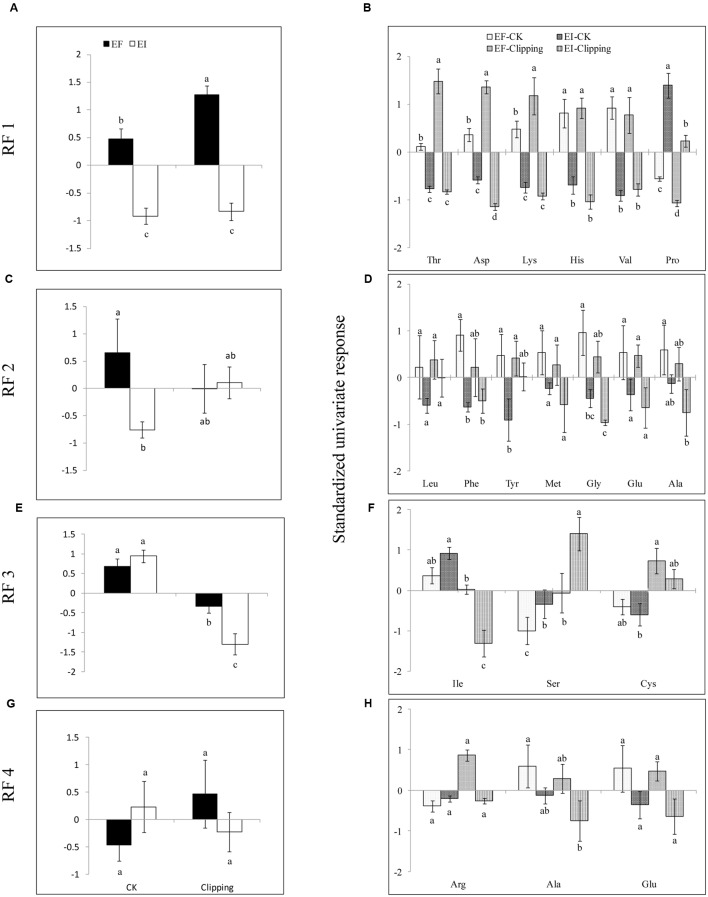
**Mean response of rotated factors (RF 1–4, A,C,E,G)** and the standardized univariate response **(B,D,F,H)** of individual amino acids in EI and EF *A. sibiricum* under unclipping (CK) and clipping treatments. Different lower-case letters denote means that are significantly different (*P* < 0.05).

The pre-column derivatization of the DNFB method and the HPLC technique were used to measure the relative composition of amino acids in the shoot of *A. sibiricum*. Because the responses of the 17 amino acids that were measured were not independent, we then used a PCA to reduce the number of amino acid response variables to a new set of composite variables. To facilitate interpretation of the principal components, we subjected the first four principal components to factor rotation with the most common form of factor rotation, varimax rotation, and we retained four rotated factors (RF1, RF2, RF3, and RF4, which accounted for 80.5% of the total variance) (**Figure 2**). As the values of the RF increased, the variables that load heavily and positively (loading ≥ + 0.5) also increased, while the variables that load heavily but negatively (loading ≤-0.5) decreased. The standardized univariate responses of these variables are shown in **Figure 3** to facilitate the interpretation of the multivariate responses and to allow a closer inspection of the variables loading heavily onto RF1, RF2, RF3, and RF4.

Five amino acids, Thr, Asp, Lys, His, and Val loaded heavily and positively onto RF1, and Pro loaded heavily but negatively onto RF1 (**Figure 2**). Endophyte infection, the clipping treatment and the interaction between endophyte infection and clipping significantly affected RF1 (**Table 2**). In the unclipped control group, RF1 in the EI plants was much lower than that in the EF plants. After clipping, there was no significant change in RF1 in the EI plants, whereas RF1 significantly increased in the EF plants. Thus, after clipping, the difference between the EI and EF plants was more obvious (**Figures 3A,B**). Leu, Phe, Tyr, Met, Gly, Glu, and Ala loaded heavily and positively onto RF2 (**Figure 2**). Only the interaction between endophyte infection and clipping (*P* = 0.088) affected RF2 under the 0.1 significance level (**Table 2**). In the unclipped group, RF2 in the EI plants was significantly lower than that in the EF plants; after clipping, however, there was no significant difference between the EI and EF plants (**Figure 3C,D**). Ile loaded heavily and positively onto RF3 while Cys and Ser loaded heavily but negatively onto RF3 (**Figure 2**). Clipping and the interaction between endophyte infection and clipping significantly affected RF3 (**Table 2**). There was no significant difference in RF3 between the EI and EF plants in the control group. After clipping, RF3 in both the EI and EF plants decreased, but it decreased more in the EI plants. Thus, after clipping, RF3 in the EI plants was much lower than that in the EF plants (**Figures 3E,F**). Arg, Glu, and Ala loaded heavily and positively onto RF4 (**Figure 2**). Neither endophyte infection nor the clipping treatment had an effect on RF4, and RF4 showed no variation before and after the clipping treatment (**Figures 3G,H**).

**Table 2:** Two-way ANOVA for rotated factors of individual amino acids of *Achnatherum sibiricum* under endophyte infection and the clipping treatment.

Treatment	Endophyte (E)		Clipping (C)		E × C	
Factor	*F*	*P*	*F*	*P*	*F*	*P*
RF1	116.0	**0.0000**	7.226	**0.0160**	4.779	**0.0440**
RF2	2.398	0.1410	0.063	0.8050	3.291	0.0880
RF3	2.953	0.1050	64.64	**0.0000**	9.343	**0.0080**
RF4	0.000	0.9970	0.276	0.6060	2.353	0.1450

*Significant P-values (P < 0.05) are in bold.*

### Soluble Sugar Content in Plants

**FIGURE 4:**
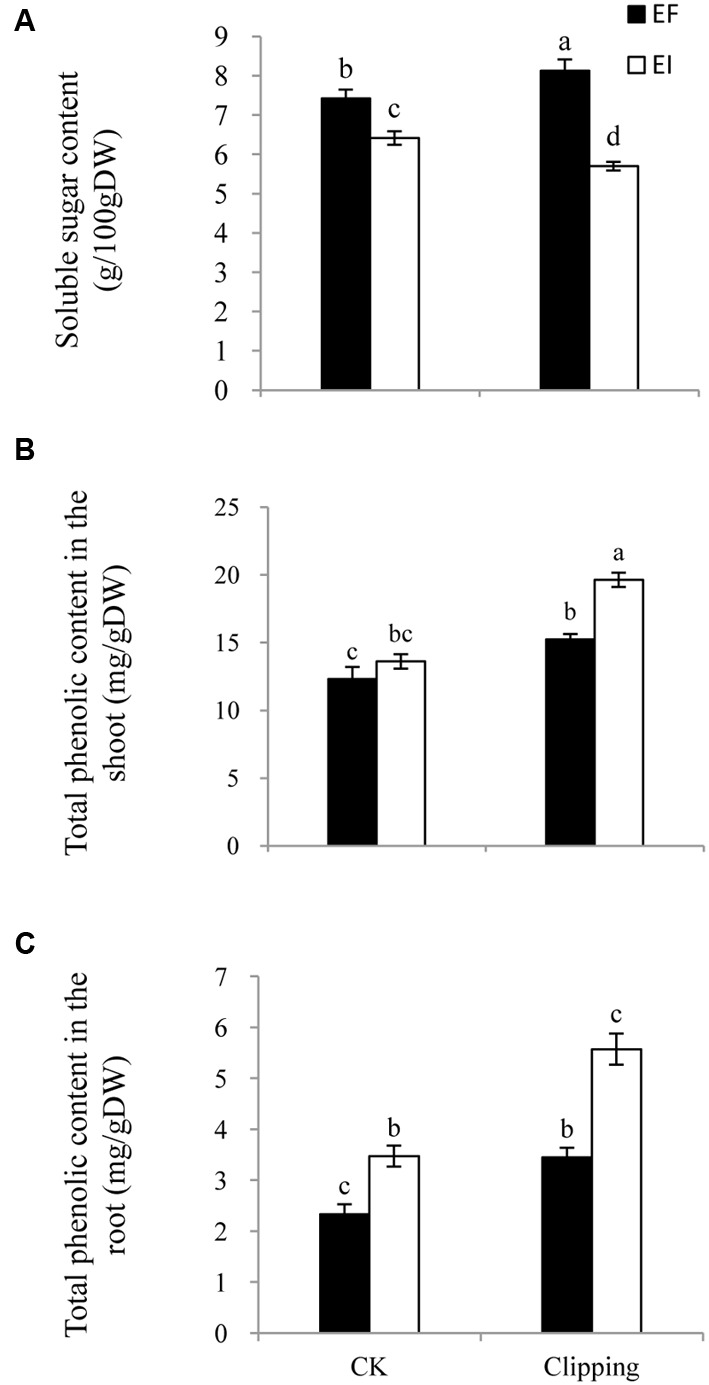
**Comparison of soluble sugar content (A)**, total phenolic content in the shoot **(B)** and the root **(C)** of EF and EI *A. sibiricum* under unclipping (CK) and clipping treatments. Different lower-case letters denote means that are significantly different (*P* < 0.05).

The soluble sugar content in the shoot of *A. sibiricum* was not only influenced by the presence of endophytes but also by the interaction of endophyte infection and clipping (**Table 1**). Endophyte infection negatively influenced the soluble sugar content. In the control group, the soluble sugar content in the EI plants was much lower than that in the EF plants. The clipping treatment increased the soluble sugar content in the EF plants but decreased that in the EI plants (**Figure 4A**).

### Total Phenolic Content in Plants

The total phenolic content of *A. sibiricum* was influenced by endophyte presence, clipping and the interaction of endophyte and clipping (**Table 1**). The total phenolic content in the shoot did not differ greatly between the EI and EF plants in the control group. After clipping, the total phenolic content increased both in the EI and EF shoots, but the EI shoots showed a greater increase (**Figure 4B**). Endophyte infection had a positive effect on the total phenolic content in the root. Clipping enhanced the total phenolic content in the root both in the EI and EF plants; similar to the total phenolic content in the shoots, the EI plants showed a greater increase than the EF plants after clipping (**Figure 4C**).

### Root Morphology and Regrowth Rate

**FIGURE 5:**
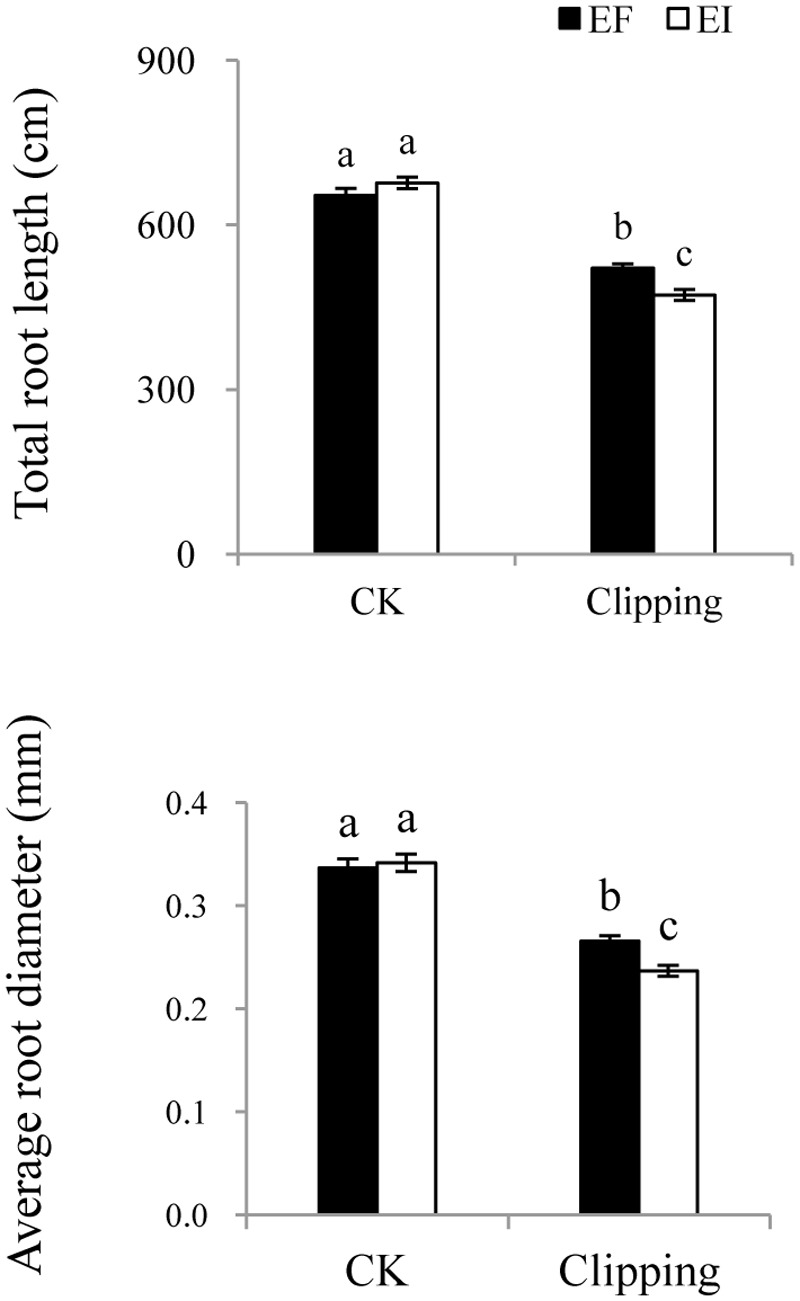
**Comparison of total root length and average root diameter of EF and EI *A. sibiricum* under unclipping (CK) and clipping treatments.** Different lower-case letters denote means that are significantly different (*P* < 0.05).

The root morphology, such as total length, total surface area and average diameter, was influenced by clipping. The total length and average diameter of the roots were also influenced by the interaction of endophyte infection and clipping (**Table 1**). The total length and average diameter did not differ greatly in the unclipped control groups. Clipping decreased the total length and average diameter of the roots in both the EI and EF plants, but those of the EI plants decreased much more than EF plants (**Figure 5**).

**FIGURE 6:**
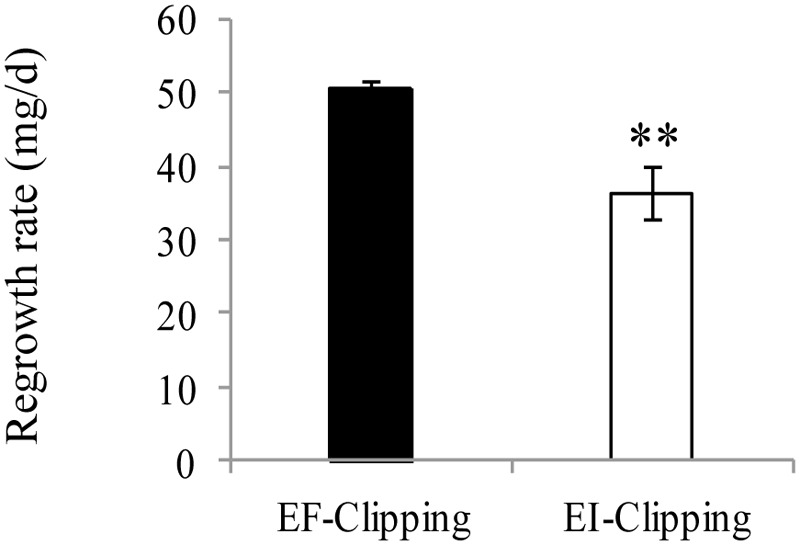
**Comparison of regrowth rate of EF and EI *A. sibiricum* under the clipping treatment.** The asterisk denotes means that are significantly different (*P* < 0.01).

To assess the effect of endophyte infection on the regrowth ability of *A. sibiricum* after clipping, we clipped the plants and allowed them to regrow for 3 weeks. We found that the regrowth rate of the EI plants was significantly slower than that of the EF plants (**Figure 6**).

## Discussion

Endophyte-conferred herbivore resistance is often attributed to the alkaloids produced by the endophyte (Clay and Schardl, 2002; Sullivan et al., 2007; Schardl et al., 2012; Saikkonen et al., 2013). For example, EI drunken horse grass can produce a certain concentration of ergonovine and ergine (Li et al., 2007), and endophyte infection could increase the resistance of the host plants to *Rhopalosiphum padi* and *Messor aciculatus*. In Arizona fescue, only a low concentration of peramine was produced, and infected plants did not show significant resistance to *Xanthippus corallipes*, *Melanoplus femurrubrum*, and *Acromyrmex versicolor* (Saikkonen et al., 1999; Tibbets and Faeth, 1999). Shymanovich et al. (2015) observed different aphid resistance of sleepy grass in different populations, with very low survival rates for the aphids feeding on plants infected with the Cloudcroft endophyte, whereas aphid survival was better on EI plants in the Weed population. In addition, they suggested that the alkaloid ergonovine was responsible for aphid mortality. In our experiments, alkaloids in neither infected nor uninfected plants were detected when grown under normal conditions. After clipping, only peramine was detected in a small number of samples in the leaf sheath of infected plants’, but the concentration was extremely low. However, we found that infected *A. sibiricum* had significant resistance to *L. migratoria*, which suggests that apart from endophyte-conferred alkaloid defense, additional mechanisms such as endophyte-mediated changes in host defense are likely to be implicated in endophyte-host-insect interactions. Similar results have been reported recently by Ueno et al. (2016), who found that ozone level did not affect alkaloid concentration but rather significantly affected aphid resistance of infected *Lolium multiflorum*.

Primary metabolites such as carbohydrates and amino acids are two important macronutrients that influence animal survival, growth and reproduction (Karasov and Martiìnez del Rio, 2007; Simpson and Raubenheimer, 2012; Roeder and Behmer, 2014). The mouthparts of locusts contain large numbers of sensilla groups with neurons sensitive to a range of chemicals, including amino acids and carbohydrates (Chapman, 2003). In the present study, we found that endophyte infection significantly decreased the soluble sugar content and amino acid content of the host plants, suggesting that endophyte infection may increase locust resistance by lowering the palatability of host plants.

In addition to alkaloids, other secondary metabolites such as phenols have been proposed as antifeedants or digestibility reducers (Ballare et al., 1996; Izaguirre et al., 2007). In the present study, we found that endophyte infection significantly increased the total phenolic content in both the shoots and roots of the host plants. Similar results have been reported in perennial ryegrass (Rasmussen et al., 2008; Pańka et al., 2013) and tall fescue (Malinowski et al., 1998). Here, the higher phenol concentration of the EI leaves may have contributed to the higher locust resistance of the host plants. Combined with the reduction of primary metabolites such as carbohydrates and amino acids, our results suggest that endophyte infection triggers reprogramming of the host metabolism, favoring secondary metabolism at a cost to primary metabolism (Dupont et al., 2015).

Plants can alter their metabolism in response to environmental conditions, such as soil nutrients, water levels and feeding by insects or mechanical damage (Bultman et al., 2004; Behmer, 2009; Machado et al., 2015). Herbivore attacks, for instance, alter nitrogen and carbon dynamics (Arnold and Schultz, 2002; Appel et al., 2012), which often results in dramatic changes in primary and secondary metabolite pools (Skrzypek et al., 2005; Steinbrenner et al., 2011; Machado et al., 2013). In particular, endophyte infection can affect the metabolism response of the host plants. For example, Bultman and Bell (2003) found that the total protein N in uninfected tall fescue significantly increased after clipping, but this was not true for infected plants. Sullivan et al. (2007) found that after mechanical and herbivore damage, uninfected tall fescue also had a significantly higher foliar N% and lower C: N ratio compared with infected hosts. In our study, we found that after clipping, the concentration of soluble sugar and Ile significantly decreased in infected plants, whereas the concentration of total phenolic significantly increased. For uninfected plants, however, clipping caused an elevation of soluble sugar and amino acid such as Thr, Lys, His, and Val. Clipping also resulted in an increase of the total phenolic content in uninfected plants, but the degree was lower than in infected plants. The clipping treatment made uninfected plants more sensitive, whereas infected plants became more resistant to the locust. The weight of the second instar locusts fed on uninfected plants increased significantly after clipping, whereas it decreased when fed on infected plants. Thus, advantage of endophyte infection in resistance to locusts was more obvious after clipping.

In addition to resistance, endophyte infection might influence the tolerance of host plants (Belesky and Fedders, 1996; Cheplick, 1998; Bultman et al., 2004). Tolerance is the degree to which a plant can regrow and reproduce following damage (Strauss and Agrawal, 1999). Hence, it is of interest to ask how endophytes influence the ability of a plant to tolerate herbivory. In tall fescue, Bultman et al. (2004) found that clipping induces resistance in infected plants at the cost of tolerance. In our study, we also found that the regrowth rate of infected plants was significantly lower than that of uninfected plants after clipping.

Endophyte infection significantly affects both primary and secondary metabolism of its host plant (Rasmussen et al., 2008). It is possible that some of the herbivore resistance effects observed in infected plants are due to the metabolites measured in this study or other, still completely unknown, endophyte-specific compounds. Therefore, wider metabolic studies are needed to understand herbivore resistance of this association.

## Conclusion

Under normal conditions, endophyte infection did not induce detectable alkaloid production, but endophyte infection did significantly enhance the resistance of the host to *L. migratoria*. In this study, the lower content of soluble sugars and amino acids and higher total phenolic content may contribute to higher locust resistance of the host plants. Endophyte infection can mediate the strategies of *A. sibiricum* response to clipping. After clipping, the infected plants exhibited decreased nutrient content, increased defense substances, and thus increased resistance to *L. migratoria* at the cost of regrowth. For uninfected plants, however, clipping caused an increase in nutrient substances and regrowth rate, but uninfected plants were more susceptible to *L. migratoria*.

## Author Contributions

Conceived and designed the experiments: AR; performed the experiments: JQ, YG, HL, YZ; analyzed the data: JQ, AR; contributed reagents/materials/analysis tools: YG. Wrote the paper: AR.

## Conflict of Interest Statement

The authors declare that the research was conducted in the absence of any commercial or financial relationships that could be construed as a potential conflict of interest.
